# Evaluation and selection of the best artificial lift method for optimal production using PIPESIM software

**DOI:** 10.1016/j.heliyon.2024.e36934

**Published:** 2024-08-27

**Authors:** Maysam Janadeleh, Reza Ghamarpoor, Nabeel Kadhim Abbood, Seyednooroldin Hosseini, Hasan N. Al-Saedi, Ali Zeinolabedini Hezave

**Affiliations:** a Department of Petroleum Engineering, EOR Research Center, Omidiyeh Branch, Islamic Azad University, Box Post: 164, Omidiyeh, 63731-93719, Iran; b Department of Petroleum Engineering, Faculty of Engineering, University of Garmsar, Garmsar, Iran; c Basrah University for Oil and Gas, Basrah, Iraq; d Department of Petroleum Engineering, Al-Amarah University College, Missan, Iraq; e Department of Management, Bahonar Technical and Engineering College of Shiraz, Fars Branch, Technical and Vocational University, Tehran, Iran; f Incubation Center of Fars Science and Technology Park, Fanavri Makzan Azma Yaran Company, Shiraz, Iran; g Incubation Center of Arak Science and Technology Park, Fanavari Atiyeh Pouyandegan Exir Company, Arak, Iran

**Keywords:** Artificial lift, Production optimization, Nodal analysis, Oil wells, PIPESIM software, Electric submersible pump (ESP)

## Abstract

A well can be produced and exploited when it has production power, in other words, if the well does not have enough pressure, it will not be able to flow. Artificial production is a method to increase the lifespan of well production. The well studied in this article has a significant annual lower pressure drop; So now with the current pressure of the existing well, it is not possible to send the oil of this well to the separator of the first stage of the exploitation unit. Among the existing solutions to maintain or increase production and increase the flow pressure of the well, is the use of artificial extraction. In this article, an attempt has been made to simulate the effect of using a core pipe, gas pumping and installing an electric submersible pump (ESP) on the production flow rate and flow pressure of the well by using the well data and the static and current pressure test. The current production data with the PIPESIM software was checked, and then the best extraction method for the studied well. Finally, the installation of an ESP was determined and selected as the best method of artificial extraction.

## 1 Introduction

Production from oil wells means the transfer of fluid from the reservoir to the well and finally to the surface of the earth [[1], [2], [3], [4], [5], [6]]. Due to high energy, some reservoirs have the ability to transfer fluid at a high flow rate and high pressure to the surface; but in some reservoirs, due to low energy or long life of the reservoir, which causes a pressure drop, an increase in the percentage of water and gas produced, the production is reduced or even stopped [[7], [8], [9]]. When the flow pressure at the bottom of the well is not enough to transfer the flow of oil to the surface with the desired amount, the natural flow of the reservoir should be enhanced by artificial production [[10], [11], [12], [13]]. In fact, artificial lift is a method to increase the productive life of the well, and as the optimal extraction method, it reduces the minimum pressure required at the bottom of the well for production and increases the extraction from the reservoir [[14], [15], [16], [17]].

Mohamed et al. [18] investigated the deepest injection points in four different wells. The results showed that the injection rate in these wells is between 1 and 10 mmscf/day. Also, the best oil recovery was obtained with the injection rate and the optimal gas injection pressure for the A-12BT2 well. Finally, it was found that increasing the pipe size increases the oil flow rate. Yadua et al. [19] developed a new model against a wellbore performance simulator with respect to the Niger Delta and the North Sea. The results showed that the average absolute deviation (AAD) was 2.7 and 5.4 % against the commercial simulator and field results, respectively. Also, the new model positively responded to its workflow, robustness, and usability. Salaudeen et al. [20] used non-commercial software to optimize the oil production system in the Nigerian Delta region located in the Gulf of Guinea. The results showed that the gas lift to the optimal system configuration increased the operating oil rate to a production rate of 1287 STB/day.

Engineers use the well simulator software that is developed and completed every day to simulate the conditions that should be implemented in the wells by spending high costs and by providing different conditions and comparing the obtained results, they choose the best option among the available options [[21], [22], [23]]. One of the available software for simulating fluid flow in wells and pipelines is PIPESIM software [[24], [25], [26]]. Today, this software offers a lot of help in how to use new knowledge and technology in the production and exploitation sector [27].

In this article, the production system was investigated using the data of the reservoir and well of one of the Iranian oil fields. Also, by using specialized PIPESIM software, the initial performance of the well was investigated in terms of production. Then, according to the characteristics of the well, the appropriate artificial lifting method was chosen and the optimal conditions were applied to it. In the next step, with the final analysis of the well, the increase in production in the well was determined. Herein, the information on one of the oil fields in southwest Iran (Yaran field) was used.

## 2 Field of study

**Fig. 1:**
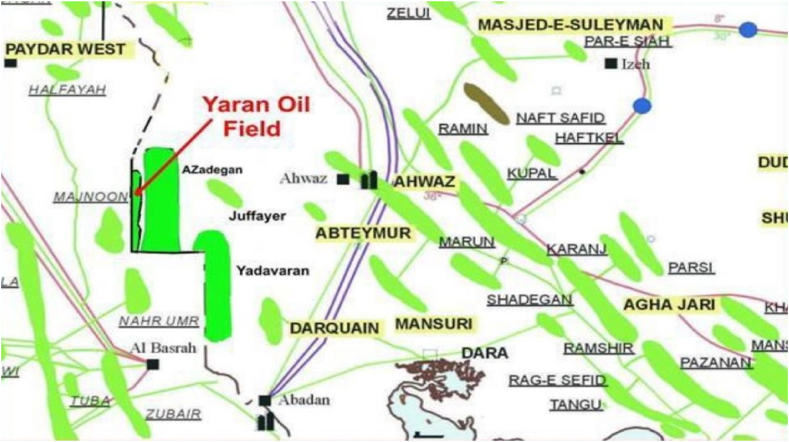
Location of the studied onshore field in SW, Iran. Reprinted in part with permission from Ref. [31]. Copyright 2021 Springer.

Yaran anticline was a geophysical anticline in the Dasht Abadan area, which is located in the vicinity and parallel to the Iran-Iraq borderline and in the west of the Azadegan oil field. The extension of this anticline is in the north-south direction and has the same trend as the Darkhovin anticline. The area of this field is about 110 (km)^2^. Yaran exploratory well No. 1 was drilled in 2018 in order to evaluate the hydrocarbon potential of the Bangistan and Khami formations and also to investigate the expansion of the Azadegan oil field. By conducting three test layers in the Fahlian, Gadwan and Sarvak formations, the presence of oil and gas with good potential in the Sarvak formation was proved. The development of this field was divided into two parts, the northern and southern Yaran (see Fig. 1) [[28], [29], [30], [31]].

## 3 Methodology

**Fig. 2:**
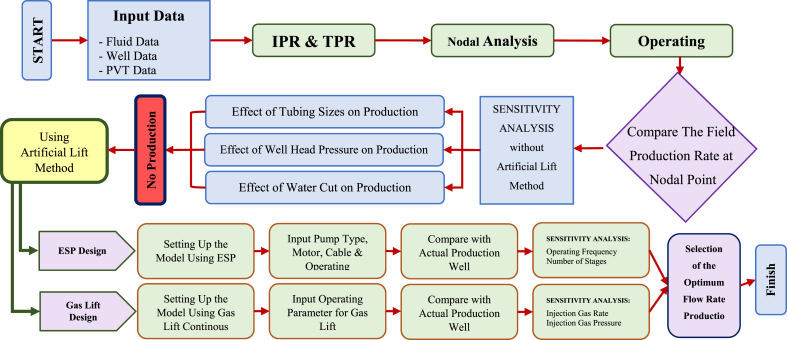
Flowchart of simulation design.

The simulation steps were carried out as follows based on the flowchart shown in Fig. (2).

1. Modeling wells under natural flow conditions to accurately understand their conditions and use them as the basis for artificial lift design.
2. Conducting sensitivity analysis on model parameters under natural flow conditions to examine the impact of parameter changes on future well production and the need for artificial lift implementation.
3. Designing and adjusting the parameters of the chosen lift method using the model developed under natural flow conditions and selecting appropriate industrial equipment for the well under investigation.
4. Conducting sensitivity analysis on specific artificial lift parameters to assess the stability and efficiency of chosen industrial equipment in response to variations in well conditions.

## 4 Well conditions

The oil well under study is located in a southwest oil field in Iran. Table 1 display the production fluid properties and well-completion details. The minimum pressure needed for flow to the operating unit is 300 psia; Calculations were performed in the Black Oil model, and the well depth was 12,000 feet. In this article, the impact of various parameters on the performance of well flow pressure variation and consequently its discharge was examined, and the optimal scenario was proposed.

**Table 1:** Production fluid properties.

Parameters	Unit	Well A
Reservoir Pressure [Pr]	Psia	3500
Reservoir Temperature [Tr]	F	200
Water Cut	%	30
GOR	SCF/STB	500
Oil Gravity	API	39
PI	STB/d/Psi	2

**Fig. 3:**
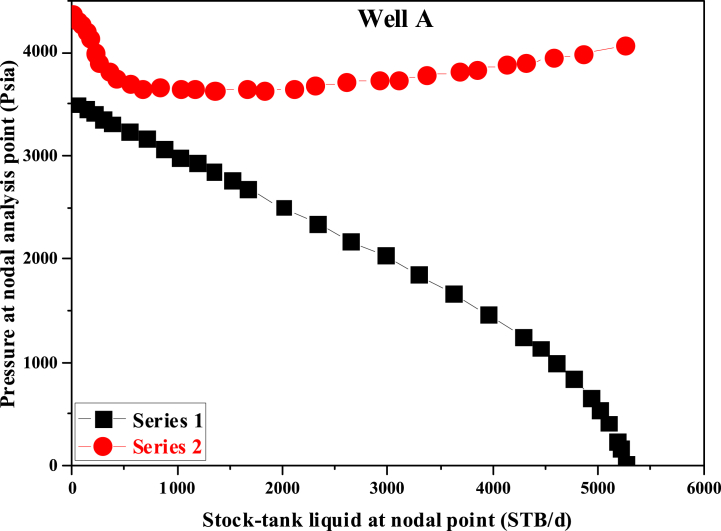
Nodal analysis under natural production conditions.

After entering the reservoir and well information, the software generates flow diagrams for both the reservoir and the well. The intersection point of these diagrams indicates the production rate of the studied well under the specified conditions. These two graphs were crucial outputs from the PIPESIM software. The simulation results indicate that the natural production of the well has been completely shut off, resulting in zero production. As shown in Fig. 3, the inflow performance relation (IPR) curve does not intersect with the tubing performance relation (TPR) curve, indicating insufficient pressure in the reservoir to bring oil to the surface and practically oil is not produced from the well. The reservoir pressure was insufficient to lift the oil to the surface, causing the hydrocarbon fluid column to remain suspended in the well, leading to no production. Based on Fig. 3, this well requires over 600 psia of pressure to enable oil extraction and initiate primary production. If we pay attention to the distance between the IPR and TPR curves on the graph, it can be observed visually that the minimum pressure required for these two curves to intersect is approximately 600 Psia, so that the desired well reaches production.

## 5 Well analysis under natural flow conditions

The nodal analysis method was utilized for calculations. When calculating well-related aspects, it was crucial to remember that the well is just one component of the production system. Extracting resources from the reservoir necessitates the fluid's journey through the reservoir rock and production layers, into the well, through the wellbore, and finally, into the collection system. The graphs shown in Fig. 3 correspond to the actual functioning of the well, because since 2010, due to the decrease in the well bottom pressure and the decrease in the flow rate of the produced oil, the well has actually been out of the production circuit, and it was necessary to take measures to make it more productive. During simulations, the well pressure was found to be 300 psia, while the exploitation unit pressure was 150 psia. Sensitivity analysis was performed on different key parameters to ascertain their effects on the well productivity. The key parameters included tubing size, well head pressure, and water cut.

### 5.1 CASE 1: Effect of tubing sizes on production

Considering a standard wellhead pressure (as used for well design) of 300 Psig and various sizes of casing diameter, A Sensitivity analysis was run for six different tubing sizes and a constant wellhead pressure, there was no production; this indicates that the lack of production was not as a result of wrong tubing size, thus the need for artificial lift system (see Table 2).

**Table 2:** Effect of varying tubing size on production rate.

Liquid Rate **[bbl/d]**	ID Tubing Size **[in]**	Well Head Pressure **[Psia]**
0	2.5	300
0	3	300
0	3.5	300
0	3.96	300
0	4	300
0	4.5	300

### 5.2 CASE 2: Effect of well head pressure on production

Considering the different wellhead pressures and the fixed pipe size (according to the well design) was 3.96 inches. According to the well design, the minimum wellhead pressure is 300 psig and at this point, there was no production. But when the wellhead pressure was reduced to 150 psig and 100 psig production began to take place. In the real case scenario, the wellhead pressure cannot be less than 200 psig, because this will cause the fluid to flow back to the well, thus the need for an artificial lift system (see Table 3) [32].

**Table 3:** Effect of varying wellhead pressure on production rate.

Liquid Rate **[bbl/d]**	ID Tubing Size **[in]**	Well Head Pressure **[Psia]**
0	3	300
0	3	250
0	3	200
720.497	3	150
1099.619	3	100

### 5.3 CASE 3: Effect of water cut on production

"The impact of water cut on production is significant in petroleum engineering. The wellhead pressure and tubing size are typically designated based on the well design, while the water cut can vary at different levels. At a water cut of 0 %, the oil production tends to be higher, while at 40 % or 50 % water cut levels, the production significantly decreases, despite the well being designed for a 30 % water cut. However, it is not feasible to completely eliminate water from the production stream, as water cut levels can fluctuate during production operations. Hence, the implementation of an artificial lift system becomes essential to optimize production efficiency and overall well performance" (see Table 4).

**Table 4:** Data on production rate.

Liquid Rate **[bbl/d]**	ID Tubing Size **[in]**	Well Head Pressure **[Psia]**	Water Cut **[%]**
654.4381	3	300	0
0	3	300	40
0	3	300	50
0	3	300	60
0	3	300	70
0	3	300	80

## 6 Application of the artificial lift method

Due to the inability of the well to produce effectively, there was a need to apply artificial lift methods to enhance production. As mentioned earlier, the two methods to be considered include the gas lift and electrical submersible pump method.

### 6.1 Gas lift method

**Fig. 4:**
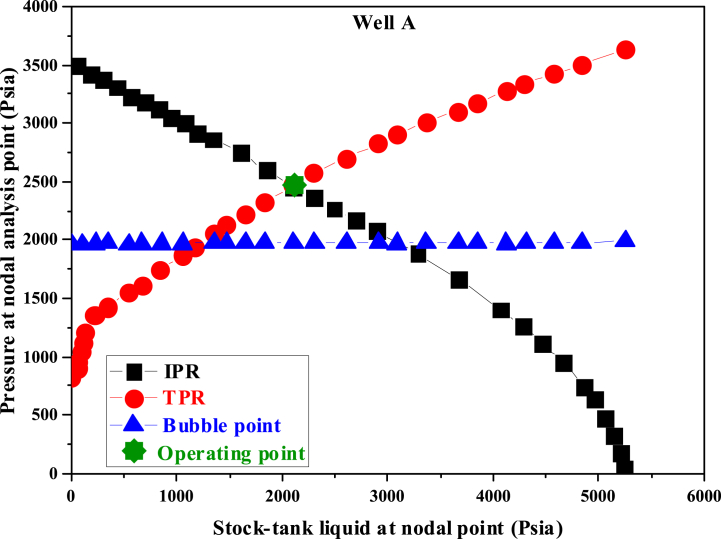
The results of the simulation of the gas lift system for well A.

In this part, the method of gas lifting in this well was investigated and the effect of different parameters on its performance was analyzed. The gas injection depth for this well was determined to be approximately 11,000 feet in the software calculations, and the majority of simulations were conducted at this depth. First, the gas lift system was simulated for this well. Fig. 4 shows the details of the gas lift system. As depicted in Fig. 4, the specific gravity of the injected gas was 0.64, with a gas injection rate of 1 million cubic feet per day, determined by the gas availability in the area. Additionally, the depth of gas injection was 11,000 feet. Also, the well's production flow rate rose from zero to 2097.81 (B/D) through the gas lift method.

The specific gravity of the gas is a crucial parameter that significantly impacts the selection of the best artificial lift method for optimal production in oil and gas wells. Specific gravity is defined as the ratio of the density of the gas to the density of air at standard conditions. It provides important information about the weight of the gas relative to air, which in turn affects the flow behavior, lifting requirements, and overall efficiency of the production system.

When evaluating and selecting the best artificial lift method using PIPESIM software, specific gravity of the gas plays a key role in determining the appropriate lifting technique, equipment sizing, and operational considerations. Higher specific gravity gases are heavier and require more force to lift to the surface, while lower specific gravity gases may be easier to handle but could pose challenges related to gas separation and fluid flow dynamics.

Specific gravity also influences the gas compressibility factor, which affects the pressure-volume-temperature (PVT) properties of the gas and impacts the performance of the artificial lift system. By considering the specific gravity of the gas in the simulation and analysis using PIPESIM software, engineers can make informed decisions regarding the selection of the most suitable artificial lift method, such as gas lift, ESP, rod pumps, or others, to optimize production and maximize recovery from the reservoir.

In conclusion, incorporating detailed information about the specific gravity of the gas in the evaluation and selection process of artificial lift methods is essential for achieving optimal production performance and efficient reservoir management.

#### 6.1.1 Sensitivity analysis to injected gas rate

Analyzing the sensitivity of well production to the injected gas rate is a crucial aspect of evaluating and selecting the most effective method for creating artificial pressure to achieve optimal production in oil fields. Through conducting a sensitivity analysis on gas injection rates, engineers can assess how variations in gas injection rates impact well productivity, wellhead production pressure, and overall production efficiency. Moreover, sensitivity analysis aids in determining the optimal gas injection rate that strikes a balance between maximizing production and minimizing operational costs.

**Fig. 5:**
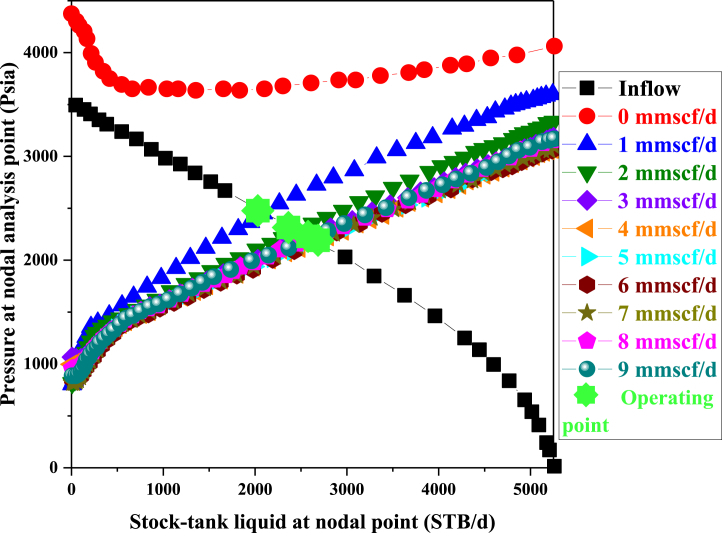
The Sensitivity Analysis with injected gas rate.

In Fig. 5, the impact of gas injection flow rate on oil production flow rate is examined. This diagram illustrates the changes in gas injection flow rate in response to variations in pressure at the Nodal Point. The analysis presented in Table 5 reveals that initially, an increase in the injected gas flow rate leads to a rise in oil production. However, this upward trend does not persist, and beyond a flow rate of 7 MMSCF/d, a decline in oil production ensues. Therefore, the optimal gas injection flow rate is determined to be 3 MMSCF/d, equivalent to 2583.106 STB/d of oil production.

**Table 5:** Effect of different gas injection rate on production.

Inj Rate **[MMSCF/d]**	ST Liq. at NA **[STB/d]**	P at NA **[psia]**
1	2097.81	2451.095
2	2455.452	2272.274
3	2583.106	2208.447
4	2634.542	2182.729
5	2660.635	2169.682
6	2667.352	2166.324
7	2661.546	2169.227
8	2645.836	2177.082
9	2622.439	2188.78

#### 6.1.2 Sensitivity analysis to surface gas injection pressure

One important factor in gas lift is the gas injection pressure at the wellhead, as it can greatly impact the method's effectiveness [[33], [34], [35]]. Table 6 illustrates how the injection pressure at the wellhead influences the performance of this operation in well A. In this table, it was evident that the ideal injection pressure is 2000 psig. Pressures exceeding this value do not impact the reservoir's production flow rate at various injection flow rates. When pressures fall below this threshold, the oil production flow rate rises as injection pressure increases. The wellhead production pressure in this segment was 300 psig.

**Table 6:** Effect of different surface gas injection pressure on production.

		Liquid Production Rate [STB/d] @ 1 Gas Injection Rate [mmscf/d]	Liquid Production Rate [STB/d] @ 2 Gas Injection Rate [mmscf/d]	Liquid Production Rate [STB/d] @ 3 Gas Injection Rate [mmscf/d]	Liquid Production Rate [STB/d] @ 4 Gas Injection Rate [mmscf/d]	Liquid Production Rate [STB/d] @ 5 Gas Injection Rate [mmscf/d]
Surface Gas Injection Pressure [psia]	1000	1969.323	2035.919	2066.358	2076.592	2073.805
	1500	2163.431	2265.733	2316.796	2341.451	2348.126
	2000	2245.526	2353.459	2395.056	2415.265	2421.672
	2500	2245.615	2353.585	2395.209	2415.412	2421.778
	3000	2245.906	2353.982	2395.727	2416.029	2422.474
	3500	2246.239	2354.436	2396.318	2416.731	2423.266

### 6.2 Electrical submersible pump method

The artificial lift pump technique is the most used in increasing oil recovery (i.e. more than 60 %), and ESPs are the most used [36,37]. ESP has a centrifugal pump, different cables and surface control [38]. These pumps convert fluid kinetic energy into hydraulic pressure [39]**.**

#### 6.2.1 Nodal analysis simulation

**Fig. 6:**
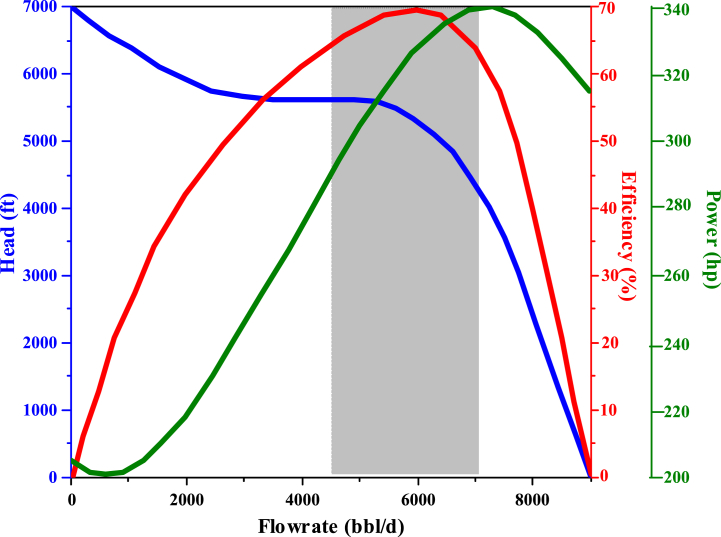
Pump performance curve of REDA D5800N (60 Hz, 235 stages).

Nodal analysis can be used to determine the flow rate and optimize artificial lift in oil and gas wells. A point that acts as a node was considered as the intersection of the input and output function curves. In this section, the goal is to increase oil production from well A to about 5000 B/D. Since REDA pumps were used in Iran, it has been tried to use this type of pump in the design. The results were obtained and the pump curve for the wellhead is shown in Fig. 6. According to the well and reservoir characteristics, desired production rate, and pump specifications, the software prioritizes pumps. Opting for the top priority ensures higher efficiency and cost-effectiveness in the long run. For well A, the optimal choice was the REDA D5800N pump. The pump curve in single-stage mode at a base speed (frequency) of 60 Hz was illustrated in Fig. 6. Experimentally, this result has been obtained that it is better to install the ESP at a distance of several hundred feet, or more precisely, about 500 feet above the perforation area, so that the production system has high efficiency and the well is more stable. As it is clear from the name of this pump, this type of pump must be placed in a floating state in the well fluid to be able to perform the pumping operation and it should not be placed close to the fluid level of the well column so that after some time the fluid level will drop below the pump. It should be noted that the deeper the pump was installed, the length of the pipe and cable, as well as the required pressure and temperature of the pump design, and the costs will increase. Therefore, according to the amount of production, fluid type, bubble point pressure, reservoir pressure drop, and the duration and amount of exploitation of the well and tank, the appropriate depth for installing the pump was considered to be 11,500 m. The pump, with a 4-inch diameter, can pump between 4400 and 7000 B/D as per Fig. 6. The well's wall diameter is 8 inches, allowing installation inside. To pump approximately 3262.103 barrels daily, the well needs 235 steps and operates at 60 Hz, achieving around 70 % efficiency. The ideal speed was 70 Hz and increasing it to 80 Hz reduces the number of steps but also lowers pump efficiency.

#### 6.2.2 Analysis of well A after installing the ESP

**Fig. 7:**
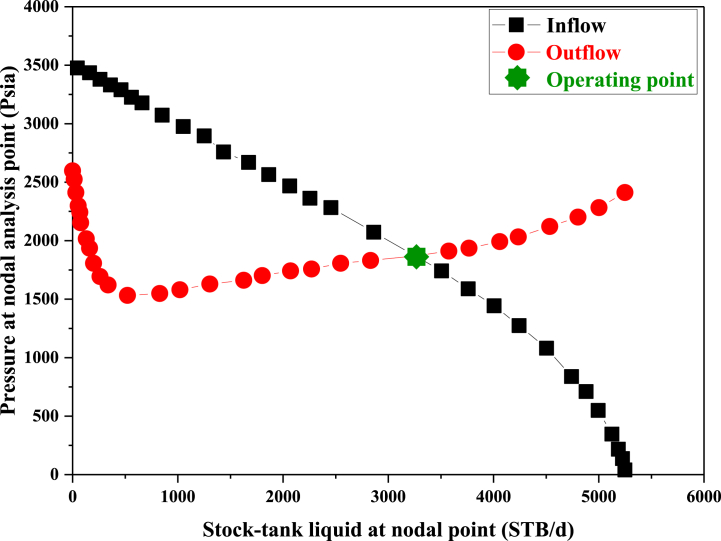
Nodal Analysis of well A after installing the ESP.

**Fig. 8:**
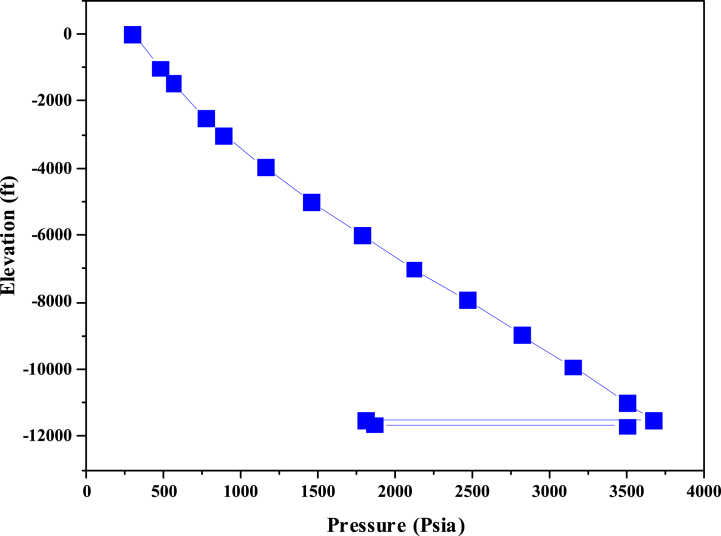
Pressure diagram versus depth of the well A after installing the ESP pump.

After installing the appropriate pump with optimal conditions for the desired well, the nodal analysis for this state of well A was investigated. In Fig. 7, the IPR curve intersects with the TPR curve, and the collision is not due to changes in the reservoir properties. The installation of the pump in the well altered the production system characteristics in the tubing, leading to a downward shift in the TPR curve, causing the collision with the IPR curve. The current production rate from the well was approximately 4000 B/D. Below is a pressure chart by depth for the well, illustrating the oil's ascent process and the pressure decline during the ascent (see Fig. 8). The pump has induced added pressure on the well system, leading to increased oil production. In well A, the pump has made it behave as if its bottom pressure exceeds 3700 psi. This alteration will elevate the production rate to 3262.103 B/D upon implementing the extraction system.

#### 6.2.3 Sensitivity analysis to operating frequency

**Fig. 9:**
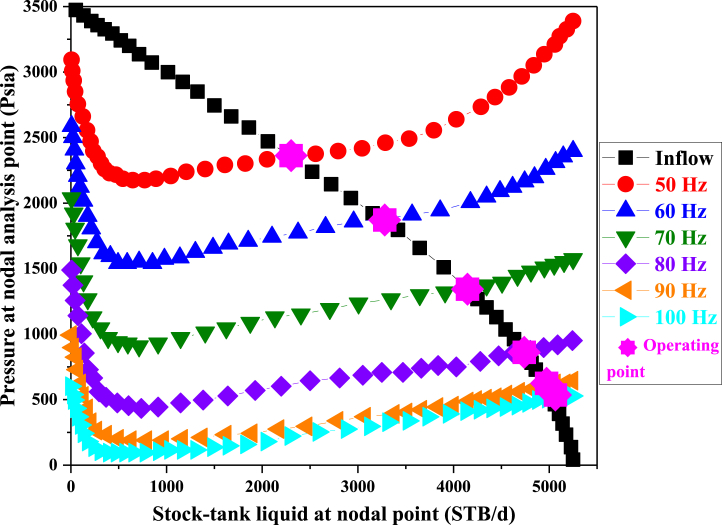
The Sensitivity Analysis with operating frequency.

In this part, the sensitivity analysis of different pump frequencies was analyzed for well A, and the result is shown in Fig. 9. In the figure below, the horizontal graph shows the rate of oil production from the well in terms of barrels per day and the vertical graph shows the rate of pressure changes at the operating point. The ideal pump frequency to achieve a daily production of around 5000 barrels for the specified well is 70 Hz. If the frequency intensity is lowered from its optimal level, production will decrease significantly. Conversely, raising the frequency intensity will initially boost oil production significantly, but beyond a certain point, the production increase will plateau. For instance, in well A depicted in Table 7, lowering the pump frequency below 70 Hz leads to a sharp production decline. Increasing the pump frequency by 30 Hz results in a substantial rise in oil production, yet beyond 100 Hz, the rate of production increase diminishes.

**Table 7:** Optimization results of operation frequency.

**Frequency [Hz]**	**ST Liq. at NA [STB/d]**	**P at NA [psia]**
50	2305.176	2347.412
60	3260.429	1868.186
70	4138.097	1347.842
80	4730.958	865.9707
90	4967.801	599.7569
100	5034.442	508.0517

#### 6.2.4 Sensitivity analysis to operating power

**Fig. 10:**
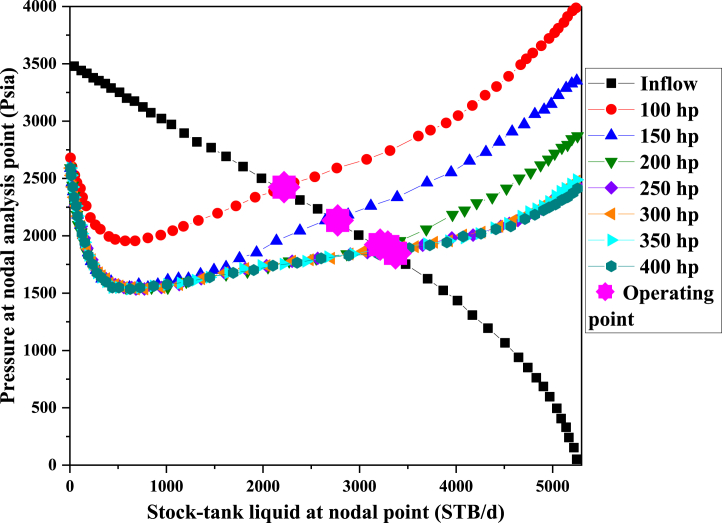
The Sensitivity Analysis with operating power.

In this part, the role of pump power changes in the desired well performance was evaluated according to Fig. 10. The optimal power of the pump to produce 5000 B/D for well A is 250 horsepower. As can be seen in Table 8, increasing the pump power from its optimal value does not affect the production rate and only causes energy wastage. But if the power of the pump decreases, it will cause a sharp drop in production.

**Table 8:** Optimization results of operation power.

Operating power **[hp]**	ST Liq. at NA **[STB/d]**	P at NA **[psia]**
100	2178.695	2410.652
150	2737.356	2131.322
200	3206.315	1896.132
250	3262.502	1867.108
300	3262.502	1867.108
350	3262.502	1867.108
400	3262.502	1867.108

## 7 Conclusion

In this study, an artificial lift system was used to investigate a well in the Yaran oil field with the aim of increasing oil production from the well. To achieve this objective, nodal analysis method was employed along with well and reservoir data to design well A using PIPESIM software. Overall, the studies in this article yielded the following results.

• Considering the production flow rates of this field and the greater flexibility of electrical submersible pumps compared to other pump types in terms of flow rate and depth, they are a suitable option for artificial lift for most wells in this field.
• Due to the high cost of gas compression for injection and the limitations in injection pressure, the use of gas lift has a lower priority compared to ESPs.
• After analyzing well A, the optimal choice is a pump operating at 70 Hz with 235 stages, boosting the well's production to 4138 barrels per day.
• The utilization of appropriate extraction systems, along with optimizing and enhancing well production, enables initial stage separation processing in the long term, which improves the quality of the produced oil.
• Based on the results and unique properties of ESP pumps that include the ability to pump high volumes of fluid, Low service and maintenance requirements, Minimal surface equipment needed, Ability to install in deviated wells and Good performance in offshore wells and the limited number of wells experiencing pressure drop, utilizing an ESP is more favorable than gas lift.
• To complete the studies, more indicators (such as production fluid characteristics, well data, tank information, and well facilities) from the fields under study can be considered.

## “Data availability statement”

All data generated or analyzed during this study are included in this published article.

## CRediT authorship contribution statement

**Maysam Janadeleh:** Methodology, Investigation. **Reza Ghamarpoor:** Writing – review & editing, Writing – original draft, Validation, Methodology, Investigation, Data curation, Conceptualization. **Nabeel Kadhim Abbood:** Writing – original draft. **Seyednooroldin Hosseini:** Supervision, Project administration, Conceptualization, Resources. **Hasan N. Al-Saedi:** Writing – original draft. **Ali Zeinolabedini Hezave:** Writing – original draft.

## Declaration of competing interest

The authors declare that they have no known competing financial interests or personal relationships that could have appeared to influence the work reported in this paper.
